# The Central Committee's Nurse Registration Bill

**Published:** 1919-05-17

**Authors:** C. E. Leo Lyle


### THE EDITOR'S LETTER-BOX—(*continued*).

### The Central Committee's Nurse Registration Bill.

*To the Editor of* THE HOSPITAL.

SIR,—May I, through your columns, be permitted to state that I have received a Representation signed by 2,318 Scottish nurses, who strongly object to certain amendments agreed to in Committee on the above Bill for the State Registration of Nurses, which is at present before Parliament, more especially to that amendment referring to Clause 12, paragraph 4, as well as to the powers given to the Provisional Council.

Clause 12, paragraph 4, accepts the Registration of a nurse if she "produces evidence satisfactory to the Council of having been for at least three years in *bona-fide* practice as a nurse in attendance upon the sick, and as to the conditions under which she was so engaged."

The nurses who have communicated with me not unnaturally object to the admission to the First Register of any person without some standard of training, and they emphasise the fact that as much as they want registration they yet realise that a bad Bill would be infinitely worse than no Bill at all, and the promoters of the present measure by agreeing to the amendments so strongly objected to clearly show that they do not represent the opinion of the great majority of the nursing profession.

The Bill, as it now stands, does not meet the needs, wishes, or views either of the trained nurses themselves or of those in course of training, but we are hoping that these objectionable features may yet be removed.—

Yours faithfully,

C. E. LEO LYLE, M.P. (Stratford).

